# Comparison of HER2 and Phospho-HER2 Expression between Biopsy and Resected Breast Cancer Specimens Using a Quantitative Assessment Method

**DOI:** 10.1371/journal.pone.0079901

**Published:** 2013-11-21

**Authors:** Yalai Bai, Huan Cheng, Jennifer Bordeaux, Veronique Neumeister, Sudha Kumar, David L. Rimm, David F. Stern

**Affiliations:** Department of Pathology, Yale University School of Medicine, New Haven, Connecticut, United States of America; Dartmouth, United States of America

## Abstract

**Background:**

HER2/Neu (ErbB-2) overexpression, which occurs in 15–20% of breast cancer cases, is associated with better response to treatment with the drug trastuzumab. PhosphoHER2 (pHER2) has been evaluated for prediction of response to trastuzumab. Both markers are heterogeneously detected and are potentially subject to loss as a consequence of delayed time to fixation. Here, we quantitatively assess both markers in core needle biopsies (CNBs) and matched tumor resections to assess concordance between the core and the resection and between HER2 and pHER2.

**Methods:**

A selected retrospective collection of archival breast cancer cases yielded 67 cases with both core and resection specimens. Both HER2 and pTyr^1248^HER2 were analyzed by the AQUA® method of quantitative immunofluorescence on each specimen pair.

**Results:**

Both HER2 immunoreactivity (P<0.0001) and pTyr^1248^HER2 immunoreactivity (P<0.0001) were lower in resections relative to CNB specimens. However, clinical implications of this change may not be evident since no case changed from 3+ (CNB) to negative (resection). Assessment of pTyr^1248^HER2 showed no direct correlation with HER2 in either CNB or resection specimens.

**Conclusions:**

The data suggest that measurement of both HER2 and phospho- Tyr^1248^HER2, in formalin-fixed tissue by immunological methods is significantly affected by pre-analytic variables. The current study warrants the adequate handling of resected specimens for the reproducible evaluation of HER2 and pHER2. The level of pTyr^1248^HER2, was not correlated to total HER2 protein. Further studies are required to determine the significance of these observations with respect to response to HER2 directed therapies.

## Introduction

Immunohistochemistry (IHC) as a method to measure HER2 expression is a standard part of the assessment of breast cancer specimens. However, the standard methods used to measure HER2 are only semi-quantitative and the standard scoring system is an ordinal, subjective score based on intensity of staining at the membrane in at least 30% of cells [1]. Although the standardization of the methods of analysis published by the American Society for Clinical Oncology/College of American Pathologists committee is helpful, there is still some degree of non-reproducibility in practice. For example, one key study showed a discordance rate of 18.4% between local and central laboratory findings [2]. Other studies have confirmed this discordance rate, including a prospective study showing that up to 20% of HER2 tests were not reproducible [1]. These observations raised questions about the source of discordance and the relative contributions of true tumor heterogeneity versus artifactual variation as a result of delayed time to fixation or other technical variables. A number of studies have suggested that loss of epitope occurs when specimens are not promptly fixed. Our earlier work suggests that the HER2 epitope is not affected when delay is less than 2–3 hours [3]. However, others have suggested substantial loss of epitope when assessing longer time points [4], [5].

Epitopic loss is an even greater concern in assessment of phospho- epitopes. Numerous studies have demonstrated that phospho-epitopes can be affected by cold ischemic time [6] [4], [7]. While initial assessment of phosphoHER2 was promising with respect to prediction of response or outcome [8], that observation has not been reproduced in other cohorts. The lack of proven value of phospho-HER2 may be due to pre-analytic variables and variable loss of epitope. It may also arise from tumor heterogeneity.

Tumor heterogeneity with respect to HER2 status has been the subject of many studies. Pathologists routinely observe cases wherein regions of strongly positive membranous staining are flanked by regions with minimal staining or no staining at all. Although all breast cancer cells (and all normal breast ductal cells) express a low level of HER2, the standard assay conditions are sufficiently insensitive that normal ducts and 85% of cases appear “negative”. Thus both the assay and the biology of the tumor suggest some level of heterogeneity, with yet unknown implications for outcome. Since, in breast cancer, HER2 is biologically activated through overexpression, it is reasonable to suppose that the extent of overexpression would be associated with the magnitude of biological outputs. While it has been shown that high level expression is associated with worse outcome and better response to therapy, moderate, heterogeneous expression is also associated with outcome [9].

A number of studies have compared core biopsies with resections [3], [4], [7], [10]. The best studies do fresh stained analysis on recut tissues to avoid artifacts related to varying antibodies or laboratory practices. However, even these studies find a consistent percentage of cases with discordant results in which the CNB and resections values differ, due either to tumor heterogeneity or possibly, to epitope decay as a consequence of pre-analytic variables. Here we use the AQUA method of quantitative immunofluorescence (QIF) to assess both HER2, and its activated form, phosphoHER2, in freshly cut and freshly stained tissue samples from both tumor resection specimens and CNBs. Our goal is to quantify differences in both epitopes and determine their relationship in the context of both heterogeneity and pre-analytic variation.

## Materials and Methods

### Cohorts

Formalin-fixed paraffin-embedded (FFPE) primary invasive breast cancer tumors were obtained from 67 patients with infiltrating ductal carcinoma or Ductal Carcinoma in Situ (DCIS) of the breast who underwent core needle biopsies and subsequent surgeries at Yale University/New Haven Hospital from 2001 to 2009. Cases were not serially collected, but rather were selected to enrich in specimens that were HER2 2+ or 3+ in the clinical assay. This non-random enrichment in higher level cases was done since cases that express normal levels of HER2 by definition have less dynamic range and are thus less likely to show detectable changes resulting from differences in pre-analytic variables. FFPE tissue blocks were obtained from archives of the Department of Pathology at Yale University. All patients were treatment-naive prior to tumor resection. The median time from core needle biopsy to tumor resection was 24.5 days. The range of the interval time between biopsy and resection was from 7 to 65 days. Time from resection to formalin fixation is unknown. The tissues were analyzed in the conventional whole tissue section format. Clinical characteristics are summarized in Table 1 and 2. The tissue assessed in this study was obtained from the Yale Pathology Archives based on Yale Human Investigation Committee protocols #9505008219, #0304025173 and #0003011706. These protocols, to Dr. Rimm, allow retrieval of tissue from archives that was consented or has been approved for use with waiver of consent. The data were analyzed anonymously from preexisting patient databases and hence exempt from consent by the human studies committee.

**Table 1:** Average ranges of clinicalpathological characteristics of 67 patients diagnosed with Breast Cancer.

Characteristic	Average (Range)
Age	58 (32–89)
Specimen size (cm)	13 (3.5–35)
Tumor size (cm)	2.15 (0.2–8.5)
Grade (score)	5 (2–5)

**Table 2:** Frequencies of clinicalpathological characteristics of 67 patients diagnosed with Breast Cancer.

Characteristic	No. of patients	%
**Node status**		
Node positive	17	25.37
Node negative	50	74.63
**Histological subtype**		
IDC	64	95.52
DCIS	3	4.48
**HER2 IHC**		
Negative (0 or 1+)	15	22.39
2+	25	37.31
3+	27	40.30
**FISH**		
NA	27	38.81
Negative	32	43.28
Positive	13	17.91

Note: Percentage may not sum to 100% as a result of rounding. Abbreviation: IDC: Infiltrating ductal carcinoma; DCIS: Ductal carcinoma in situ; NA. Not applicable.

### Tissue microarrays

Additional specimens on the breast cancer index array (YTMA147) were used as the control array and analyzed in parallel with test arrays in each experiment. This allowed us the construction of a normalization standard curve to adjust for HER2 and pTyr^1248^HER2 run-to-run variability. Formalin-fixed paraffin- embedded pellets of cell lines were used as controls: SKOV3, T47D, SKBR3, BAF3, BT474, MB468, MB453, MCF7, MB175, SW480, H2126, H1666, H2279, MB436, ZR751, SUM159, BT20 and UACC812 were purchased from the American Type Culture Collection or donated by other laboratories. Culture conditions and cell-line tissue microarray construction have been published [11] [12].

### Western Blotting

Protein was extracted from cells and incubated at 100°C in Laemmli SDS sample buffer for 5 min. Equal amounts of protein per sample were separated by SDS-polyacrylamide gel electrophoresis, transferred electrophoretically to a nitrocellulose membrane (Bio-Rad), and blotted with mouse monoclonal antibodies to pTyr^1248^ HER2 (Thermo Fisher Scientific, Clone PN2A, Fremont, CA) followed by anti-mouse horseradish peroxidase–conjugated secondary antibodies (Santa Cruz Biotechnology, Santa Cruz, CA) diluted 1∶4,000 and detected with the use of enhanced chemiluminescence (SuperSignal West Pico, Thermo Scientific, Rockford, IL). β-Tubulin (rabbit polyclonal, Cell Signaling Technology, Danvers, MA) was used for comparison of total protein loading.

### Antibodies and Immunohistochemistry

Whole tissue sections and control arrays of each run were incubated at 60°C overnight prior to deparaffinization with xylene, rehydration, and antigen- retrieval for 15 min in citrate buffer (pH = 6). Slides were preincubated with 0.3% bovine serum albumin in 0.1 mol/L TBS (pH = 8) for 30 min at room temperature to block non-specific binding. Following these steps, slides were incubated with a cocktail of pTyr^1248^HER2 antibody diluted at 1∶100 (Mouse monoclonal, clone PN2A; Thermo Fisher Scientific, Fremont, CA) or HER2 antibody diluted at 1∶1000 (Mouse monoclonal, Clone CB11, Biocare Medical, Concord, CA) and a wide-spectrum rabbit anti-cow cytokeratin antibody (Z0622; Dako Corp, Carpinteria, CA) diluted 1∶100 in bovine serum albumin/TBS overnight at 4°C. This was followed by a 1-hour incubation at room temperature with Alexa 546-conjugated goat anti-rabbit secondary antibody (A11010; Molecular Probes, Eugene, OR) diluted 1∶100 in mouse EnVision reagent (K4001, Dako Corp, Carpinteria, CA). Cyanine 5 (Cy5) directly conjugated to tyramide (FP1117; Perkin-Elmer, Boston, MA) at a 1∶50 dilution was used as the fluorescent chromogen for pAKT detection. Prolong mounting medium (Prolong Gold, P36931; Molecular Probes, Eugene, OR) containing 4′,6-diamidino-2-phenylindole was used to identify tissue nuclei. Negative control sections, in which the primary antibody was omitted, were used for each immunostaining run.

### Quantitative Immunofluorescence

QIF using the AQUA method allows quantification of protein concentration within individual subcellular compartments with high reproducibility, as described previously [13], [14]. In brief, a series of high-resolution monochromatic images was captured by the PM-2000 microscope (HistoRx). For whole tissue sections, multiple fields of view (FOVs) containing only invasive tumor were selected by investigators overseen by certified pathologists using the images created by cytokeratin immunohistochemical stains to judge invasive vs in situ carcinoma, vs normal breast structures. Target protein signal was measured using a channel with emission maxima above 620 nm, in order to minimize the contribution of tissue autofluorescence. Tumor was distinguished from stromal and non-stromal elements by creation of an epithelial tumor “mask” based on localization of the cytokeratin signal. This yielded a binary mask (each pixel being either “on” or “off”) on the basis of an intensity threshold set by visual inspection of histospots. The AQUA score of the target protein in each subcellular compartment was calculated by dividing the target protein compartment pixel intensities by the area of the compartment within which they were measured. AQUA scores were normalized to the exposure time and bit depth at which the images were captured, allowing scores collected at different exposure times to be directly comparable.

### Statistical analysis

The average values for target AQUA scores from multiple FOVs were calculated and treated as independent continuous variables. The Wilcoxon Signed Rank test and Student's paired t test were employed to assess the paired differences, considered significant at a p-value less than 0.05. Error bars in the accompanying graphs represent 95% confidence interval (CIs).

## Results

### CB11 Antibody validation and HER2 detection normalization

**Figure 1:**
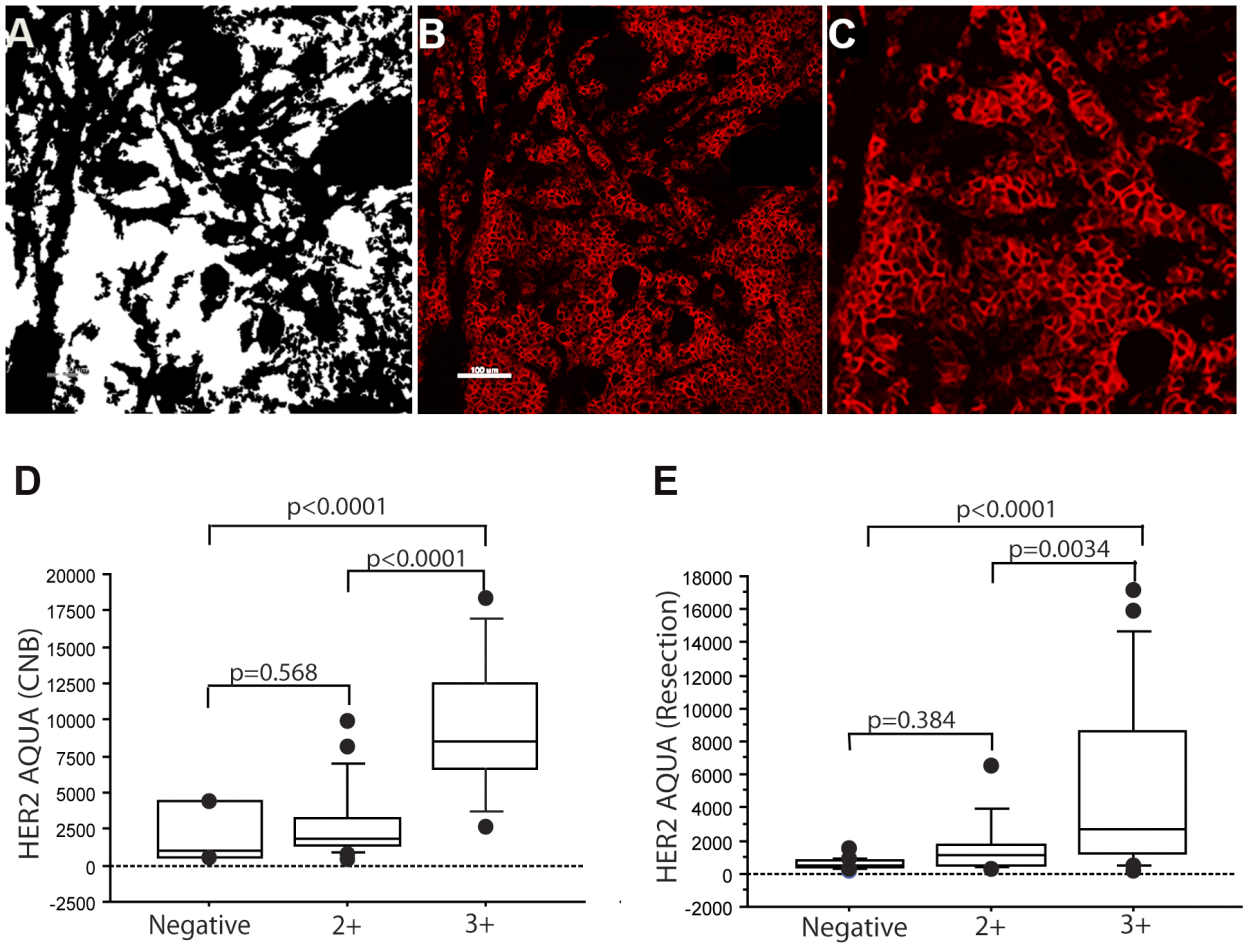
HER2 detection by immunofluorescence in control arrays, and concordance between HER2 AQUA vs. conventional HER2 IHC. A: Assignment of the tumor compartment as defined by cytokeratin and DAPI fluorescence in sections with invasive or DCIS lesions. **B**: Representative immunofluorescent staining of HER2. Original magnification, 20×. **C**: HER2 signal visualized with high magnification. **D**: box plots of the distribution of HER2 AQUA versus groups scored by clinical IHC criteria as HER2 negative, 2+ and 3+ evaluated on core needle biopsies (CNBs). **E**: box plots of the distribution of HER2 AQUA versus groups scored as clinically HER2 negative, 2+ and 3+ evaluated on resection specimens.

Monoclonal antibody clone CB11 specifically targets an epitope in the intracellular domain of HER2 and is widely used in the clinical setting to identify breast cancer patients for subsequent Herceptin treatment. Figures 1B and 1C show typical membranous staining patterns in a tissue defined as HER2 3+ by conventional clinical IHC. CB11 AQUA was positive for both BT474 and SKBR3 cell lines, which are known to over-express HER2 (data not shown). In order to minimize run to run variation, nearly consecutive sections of index tissue microarray YTMA147, which includes breast cancer tissue and breast cancer cell lines, were stained in parallel with test samples for all runs. Pearson's R ranged from 0.88 to 0.98 for YTMA147 (Fig. S1). AQUA scores of HER2 were normalized based on standard curves from the control array sections.

### Comparison of AQUA and IHC concordance in specimens with HER2 IHC assessment

AQUA assessment scores are determined from the sum of specific signals in the areas delineated by the tumor mask. Both the size of the expressing area and pixel intensities are taken into consideration [13]. In clinical settings, HER2 status is evaluated by IHC based on the percentage of HER2 positive cells and whether the membranous staining pattern is uniform and circumferential as compared to only partial or incomplete (FDA and ASCO/CAP criteria). We compared “standard” HER2 IHC results and HER2 AQUA analysis in order to evaluate their mutual consistency. In practice, the HER2 IHC status of most patients was evaluated either in biopsy or resection, while only a few cases had HER2 IHC results for both biopsies and resections. We found that in both biopsy specimens and resections where HER2 IHC had also been performed, the average AQUA scores of the group determined using standard clinical IHC criteria to be HER2 3+ are significantly higher than those of the group scored either negative for HER2 or weakly positive (Fig. 1D and Fig. 1E), indicating overall consistency between the methods.

**Figure 2:**
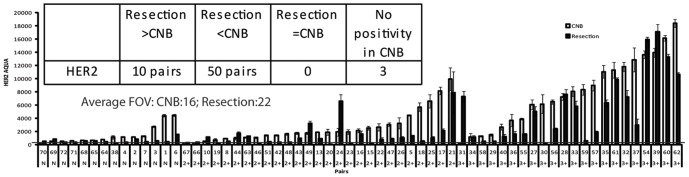
Expression of HER2 in tumor resections compared to CNBs. AQUA HER2 scores of 63 pairs of CNB (open bars) and tumor resection (filled bars) were assessed. Average FOVs of CNB: average number of FOVs included in analysis of CNB; Average FOVs of Resection: average number of FOVs included in analysis of tumor resection specimens. The noise cut point is marked with a solid line. Each AQUA score represents the mean ±95% CI. The number of pairs where resections are higher, lower than CNBs, or equal to those of CNBs are listed in inset.

63 pairs of specimens were tested by HER2 AQUA after exclusion of CNB/resection pairs having insufficient tumor area for analysis. The average AQUA score of images without specific signal (306) was chosen as the HER2 signal to noise cut point. Most of the biopsies were HER2 AQUA positive (Fig. 2). For each specimen, the maximum number of fields of view (FOV) with 20× magnification were collected to minimize influence of tumor heterogeneity. The average numbers of FOVs analyzed for the biopsies and resections were 16 and 22, respectively. For each biopsy specimen, the average HER2 AQUA score of all available fields was compared to the average of its paired resection specimen. HER2 AQUA scores of biopsies were significantly different from those of resections, with p<0.0001 in both Wilcoxon Signed Rank test and Student's paired t test (Table 3). 10 pairs had greater HER2 AQUA scores in resection than in CNB, 50 pairs had less HER2 in resections. We also compared the HER2 AQUA scores of the specimens in clinical negative (0 or 1+) group, weakly positive group and strong positive groups. There were significant differences in all groups.

**Table 3:** HER2 and pTyr^1248^HER2 AQUA score comparisons between CNBs and resections.

Marker status (# of cases)	Wilcoxon Signed Rank Test	Paired t test	Mean of Difference (CNB-R)
HER2 Overall (63)	<0.0001	<0.0001	1579.0
HER2 Group 0 or 1+ (14)	0.0067	0.0221	871.7
HER2 Group 2+ (27)	0.002	0.0066	1233.1
HER2 Group 3+ (22)	0.0077	0.008	2454.0
pHER2 Overall (35)	<0.0001	<0.0001	401.5
pHER2 Group 0 or 1+ (5)	0.1875	0.0865	364.8
pHER2 Group 2+ (14)	0.0017	0.0014	358.4
pHER2 Group 3+ (16)	0.0055	0.0143	450.7

Note: Abbreviation: CNB. Core Needle Biopsy; R: Resection.

### Comparison of phosphoHER2 expression in CNBs vs. surgical tumor resections

**Figure 3:**
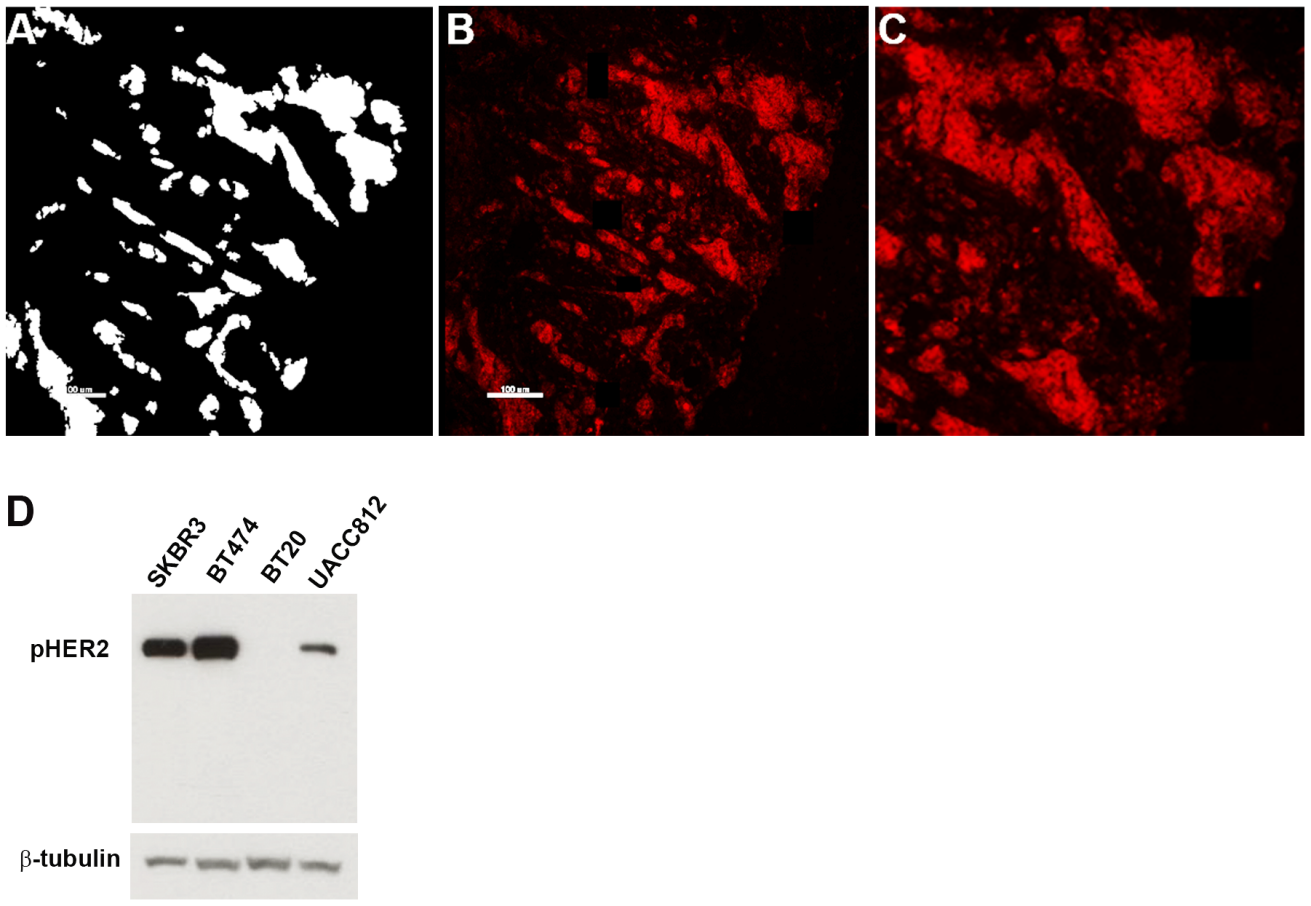
Fluorescent pTyr^1248^HER2 IHC in control arrays and antibody validation. A: Assignment of the tumor compartment as defined by cytokeratin and DAPI detection in sections with invasive or DCIS lesions. **B**: Representative immunofluorescent detection of pTyr^1248^HER2. Original magnification, 20×. **C**: pTyr^1248^HER2 with high magnification. **D**: phosphoTyr^1248^HER2 was detected by Western blot in quiescent cell cultures. β-tubulin was used as a loading control.

**Figure 4:**
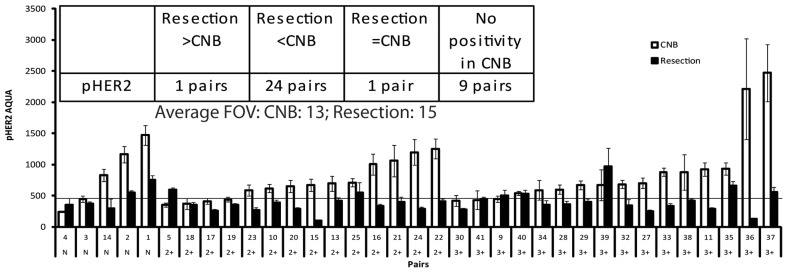
Expression of pTyr^1248^HER2 in paired specimens. A: AQUA pTyr^1248^HER2 scores for 35 pairs of CNB (open bars) and tumor resection (filled bars). Average FOVs of CNB: average number of FOVs included in analysis of core needle biopsies; Average FOVs of Resection: average number of FOVs included in analysis of tumor resection specimens. Each AQUA score represents the mean ±95% CI. The noise cut point is presented as a solid line. The number of pairs where resections are higher than CNBs, lower than CNBs, or equal to those of CNBs are listed in inset.

HER2 is tyrosine-phosphorylated on multiple sites that couple the receptor to signaling efferents. Tyrosine 1248 is a major site associated with oncogenicity and coupling to RAS/MAP kinase pathway signaling [15], [16]. Monoclonal antibody PN2A, which is specific for Tyr 1248 [15], recognizes membrane- localized HER2 (Figure 3 B and 3 C) in a subset of the clinically HER2 strong positive specimens, and also in cell lines where HER2 is known to be overexpressed and phosphorylated [17] (pictures not shown). As for HER2, near-consecutive sections of YTMA147 were analyzed in parallel to test sections for run to run normalization of pTyr^1248^HER2 (Fig. S2). Pearson's R ranged from 0.69 to 0.82. 35 randomly-selected pairs of biopsies and resections were analyzed (Figure 4). The average number of FOVs analyzed for pTyr^1248^HER2 was 13 and 15 for CNBs and resections, respectively. AQUA score 450 was determined as the cut point of signal to noise based on the average AQUA score of fields that did not have immunoreactive pTyr^1248^- HER2 (solid line). 1 pair had greater pTyr^1248^-HER2 AQUA scores in resection than in CNB, 24 pairs had less pTyr^1248^-HER2 in resections, the scores were equal for one pair (Figure 4). The average of pTyr^1248^HER2 AQUA per specimen was compared with each counterpart. Reduced levels of pTyr^1248^HER2 in resections relative to biopsies were statistically significant in mixed groups and in HER2 2+ and 3+ groups (Table 3). 13 out of 16 HER2 strongly positive CNBs (81.25%) have the activated form of HER2 marked with pTyr^1248^-HER2. Of five HER2 CNBs that were scored negative by clinical IHC, 3 are pHER2 positive by AQUA (Fig. 4).

### Bivariate comparison of pTyr^1248^HER2 and HER2

**Figure 5:**
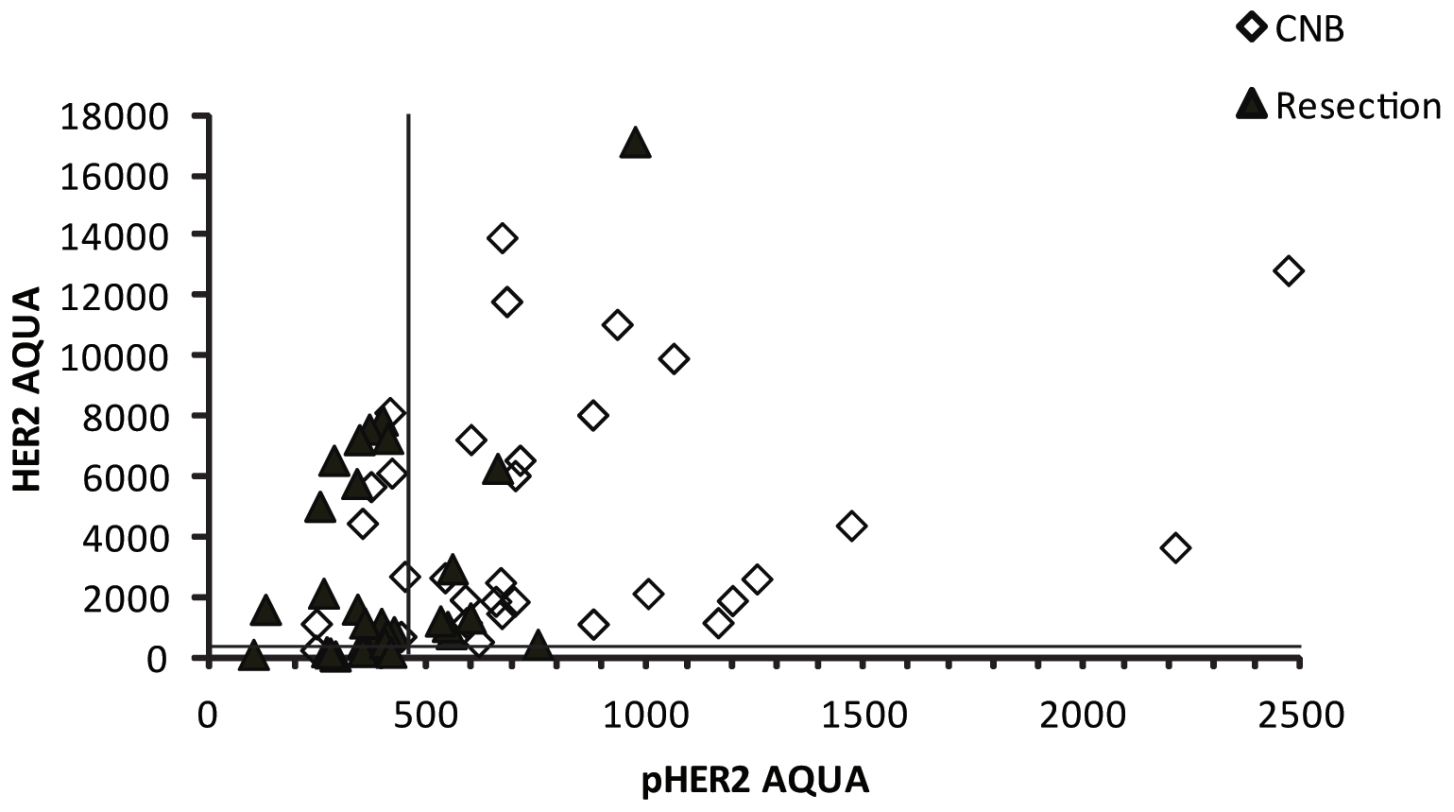
Comparison between pTyr^1248^HER2 and HER2 AQUA. Scatter plot of HER2 and pTyr^1248^HER2 AQUA scores (CNBs, open diamonds; Resections, filled triangles). Lines in the graph represent the noise cut points of either HER2 or pTyr^1248^HER2.

HER2 AQUA and pHER2 AQUA were compared to determine whether they correlate (Fig. 5). There was no significant correlation of HER2 and its phosphorylated form in either biopsies or resections. Specimens with lower levels of HER2 tended to have less pTyr^1248^HER2, and a few of the specimens scored low for HER2 by AQUA had pHER2, while specimens with high HER2 expression was associated with varying degrees of phosphorylation. Hence, immunoreactive pTyr^1248^HER2 varies somewhat independently of HER2 status.

## Discussion

Accurate assessment of HER2 is critical for therapeutic decision making. Recent controversies over trastuzumab-sensitivity of breast cancers scored by clinical IHC as low or negative reinforce the need for accurate and quantitative assessment. The literature suggests that the accuracy of assessment of HER2 expression remains uncertain [7], [18], [19], Studies by our group and others have demonstrated that the extent of protein expression and phosphorylation in formalin-fixed paraffin-embedded tissues is affected by pre-analytic variations, especially time to fixation [6], [7]. In this study, by using HER2 in CNBs as standard control, comparison of HER2 expression between resections and CNBs was quantitatively assessed, in order to determine if HER2 is labile to pre-analytic variables prior to fixation including duration of cold ischemic time.

It is not uncommon to find discrepancies of conventional HER2 IHC result between CNBs and paired resections [18]–[21]. However, these results are complicated by the inter- or intra- observer variations-, and the semi- quantitative nature of HER2 IHC. AQUA, which evaluates quantitative variables over a broad dynamic range, and scores assays quantitatively in the context of masking reduces the impact of human subjectivity [13]. The current study is the first to employ automated imaging analysis system to address the HER2 stability issue in conventional whole slide tissue specimens. Additionally, this is the first study to address the concordance between standard HER2 scoring and AQUA HER2; and the first to quantitatively assess the correlation between HER2 and PhosphoHER2 using AQUA.

Both HER2 and pHER2 were significantly lower in resections than CNBs evaluated by both Wilcoxon Signed Rank test and students paired T test (Fig. 2, Fig. 4, Table 3). Although it is conceivable that this reflects a true biological difference owing to changes in tumors between CNB and resection, it is not likely that this differential is the result of negative conversion [22], since the median time interval between the CNB procedure and tumor removing surgery was only 24.5 days, and none of the patients received treatment between biopsy and resection. Another explanation may be intratumoral heterogeneity which developed either from subclone diversity [23] [24] or from varying levels of ischemia due to poorly organized tumor blood vessels [9], [25]. Indeed, HER2 intratumoral heterogeneity may be found in 30% to 50% breast cancer cases [26] [27] [28] [29]. Although we attempted to minimize the impact of intratumoral heterogeneity by surveying as many 20× fields of view as possible for CNBs and resections, the CNBs are necessarily small. We note that 10/63 pairs and 1/25 pairs had greater AQUA scores of HER2 and pHER2 respectively, in the resection than in the CNB (Fig. 2 and Fig. 4). It is probable that intratumoral heterogeneity contributed to this observation, whereas the consistently lower levels of HER2 or pHER2 levels in resection specimens (50/63 for HER2 pairs; 24/25 pHER2 pairs) are unlikely to be explained solely by intratumoral heterogeneity. (Fig. 2 and Fig. 4). On balance, it seems most likely that technical issues are at play, especially since several technical issues are well known to contribute to signal loss. Hypoxic and ischemic conditions promote degradation and dephosphorylation of HER2 beginning with tissue disruption and attendant loss of circulation once the blood supply is interdicted [30]–[32]. CNBs offer significant technical advantages over resections since the time from sample acquisition to fixation is a matter of seconds for CNBs, and potentially hours for resections. Moreover, the narrow diameter of CNBs accelerates penetration of fixative and promotes completeness of fixation.

pHER2 antibodies were originally developed to enable measurements of HER2 signaling, rather than abundance, in tumor tissue, and they are effective in quantifying HER2 signaling in tissue culture models. However, their utility as prognostic and predictive indicators in clinical settings is uncertain. There is controversy as to whether pHER2 is associated with sensitivity of trastuzumab [33]–[35], and clinical studies using these reagents are limited in scope and scale. As discussed in earlier reports, pHER2 is expected to be loosely linked to total HER2. In breast cancer, HER2 overexpression is a major mechanism promoting activation, which is associated with phosphorylation. Moreover, the absolute number of HER2 molecules sets the maximal limit for the pHER2 signal, but within this range, the entire gamut of pHER2 signal could be expected, depending on the fraction of HER2 molecules that are active. Finally, a potential artifactual source of differential immunodetection of HER2 versus pHER2 is the greater lability of phosphoepitopes than total protein epitopes. For this reason, phospho-HER2 is expected to be more susceptible to the technical issues post harvesting of tissue that may well dictate lower signals in the resections.

One of the goals for use of pHER2 immunodetection in clinical studies was the hypothesis that HER2 may in some cases be activated (and hence a plausible therapeutic target) without HER2 amplification, for example if autocrine or paracrine agonists are present that activate EGFR, HER3, and HER4 and lead to HER2 activation in heterodimers. This may not be practical using IHC, since the cutoff for detection with conventional IHC is in the same neighborhood as the expression of HER2 in the absence of *HER2* amplification. This is the reason that normal breast tissue and non-*HER2*-amplified breast cancer general score negative for HER2 by IHC, despite the presence of more than 10,000 HER2 molecules per cell. The turnover of activated HER2 may further diminish the steady-state number of active molecules, pushing the ceiling for pHER2 detection of a single epitope even lower. In contrast, AQUA detection offers considerably greater sensitivity and dynamic range for pHER2 quantification, so that formerly sub-threshold populations of pHER2 could potentially be imaged and quantified. This may turn out to be especially important with reports that some patients determined to be HER2-negative by IHC have responded to trastuzumab (although some of these analyses may be erroneous) [36].

In our study, the phosphorylated form of HER2 did not correlate well with HER2 (Fig. 5). This result is consistent with previous studies that pTyr^1248^HER2 is not simply a surrogate of HER2 [37] pHER2 positivity was noted in some specimens without HER2 over expression, perhaps aided by greater sensitivity of AQUA detection [37], [38] (Fig. 4). One explanation is that pHER2 is induced by other members of the EGFR network including EGFR [39], so it is possible that patients without HER2 over expression could still benefit from Trastuzumab [36].

## Conclusions

In summary, this work quantitatively assesses potential effects of delayed formalin fixation and other pre-analytic variables on surgical resections by using biopsies as standardized controls. Both HER2 and pTyr^1248^HER2 showed significant reduction in expression in the resection specimens. The results indicate that conventional resection tissues with uncontrolled cold ischemic time are not optimal for companion diagnostic testing.

## Supporting Information

Figure S1 **HER2 standard curves for run to run normalization.** A, B, and C: HER2 AQUA scores of the same spots determined on serial cuts of the YTMA147 index array analyzed in parallel with test samples yield standard curves for AQUA normalization. (Pearson's R ranged from 0.88 to 0.98).
(TIF)

Figure S2 **pTyr^1248^HER2 standard curves for run to run normalization.** A, **B**, and **C**: pTyr^1248^HER2 AQUA scores of the same spots determined on serial cuts of the YTMA147 index array analyzed in parallel with all runs yield standard curves for AQUA normalization. (Pearson's R ranged from 0.69 to 0.82).
(TIF)
