# Supplementary figures and images for: Comparison of HER2 and Phospho-HER2 Expression between Biopsy and Resected Breast Cancer Specimens Using a Quantitative Assessment Method
Source: PLoS One. 2013 Nov 21;8(11):e79901. doi: 10.1371/journal.pone.0079901 (PMC3836903; doi:10.1371/journal.pone.0079901)

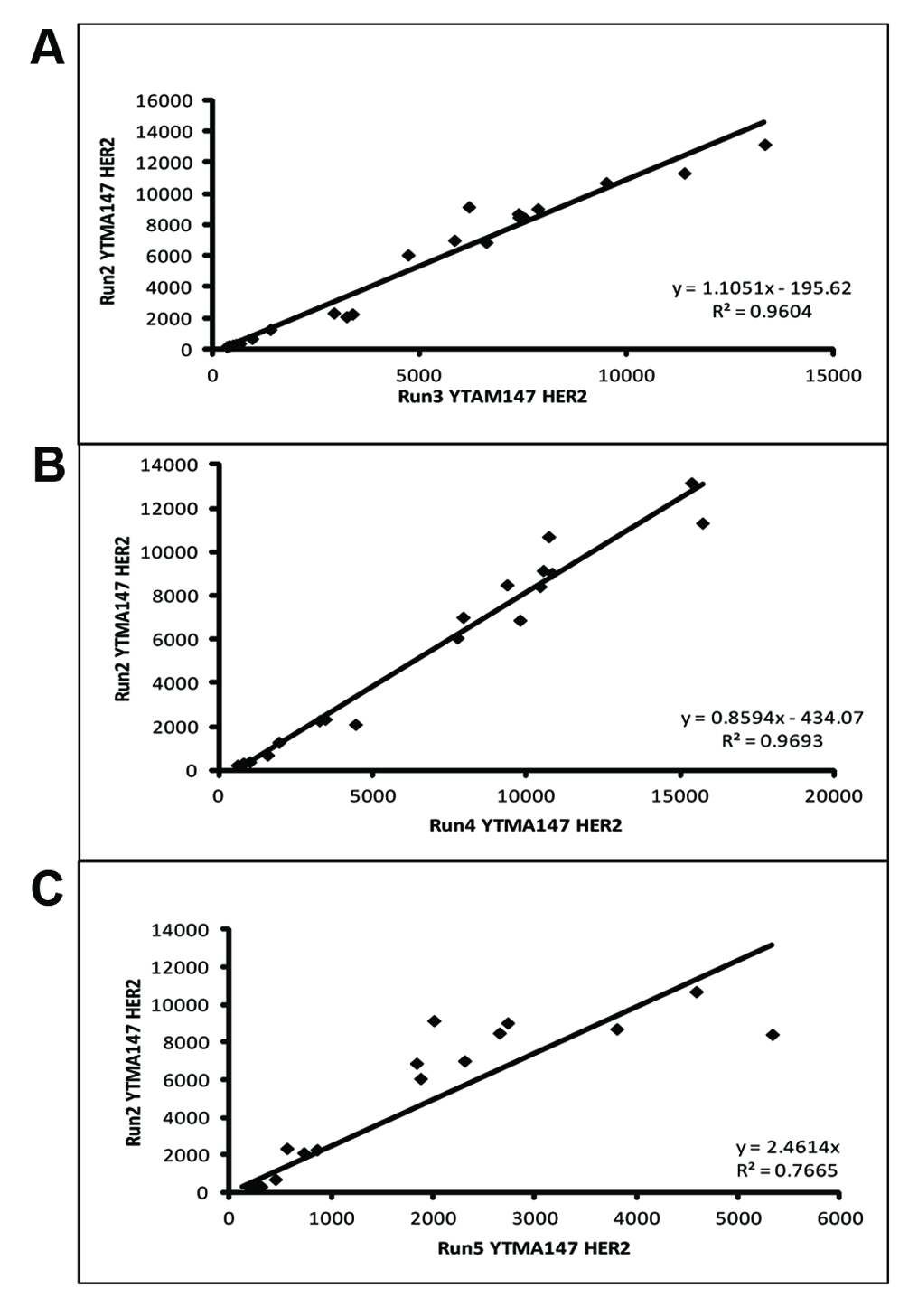

Supplement: Figure S1 — HER2 standard curves for run to run normalization. A, B, and C: HER2 AQUA scores of the same spots determined on serial cuts of the YTMA147 index array analyzed in parallel with test samples yield standard curves for AQUA normalization. (Pearson's R ranged from 0.88 to 0.98). (TIF) [file pone.0079901.s001.tif]

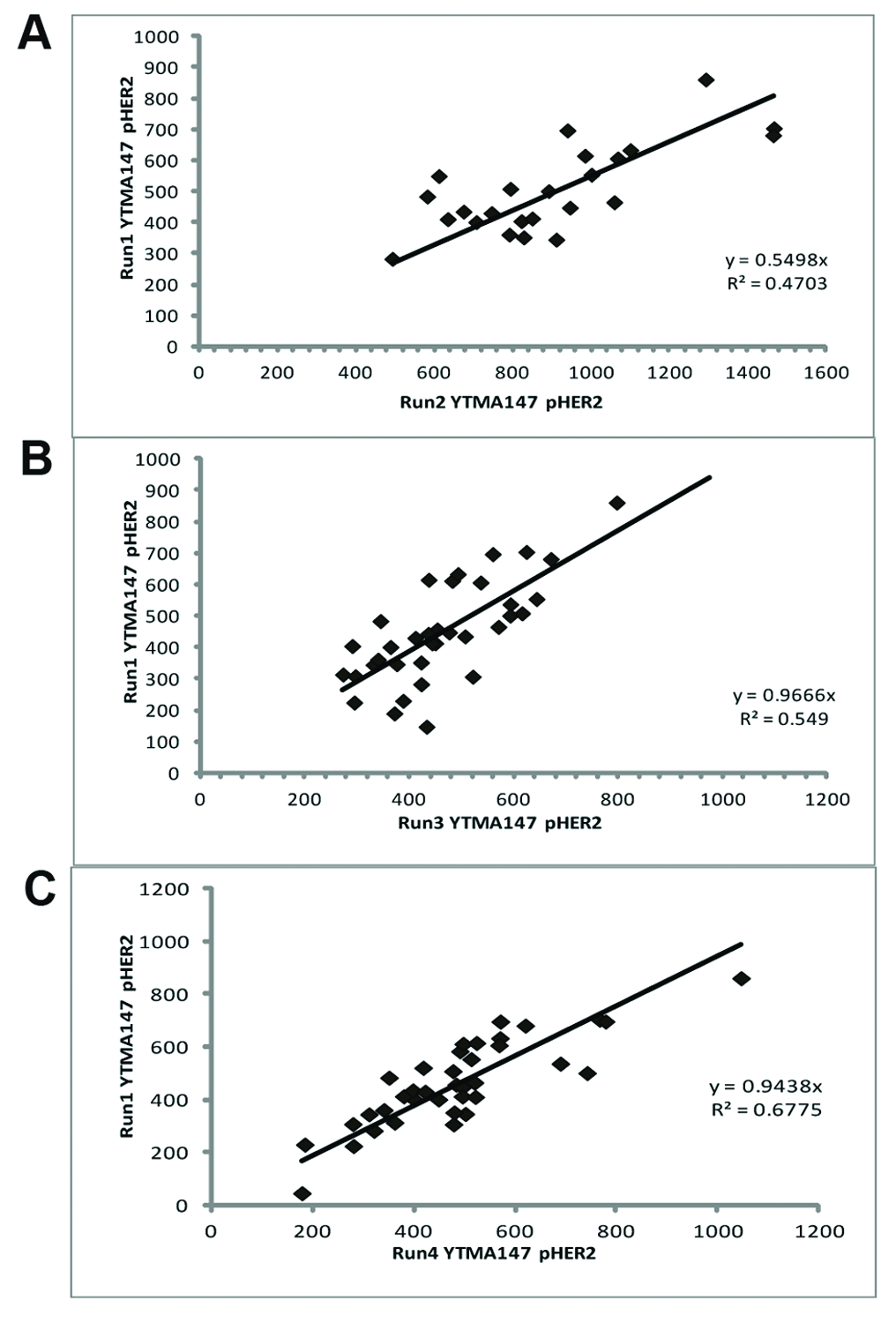

Supplement: Figure S2 — pTyr1248HER2 standard curves for run to run normalization. A, B, and C: pTyr1248HER2 AQUA scores of the same spots determined on serial cuts of the YTMA147 index array analyzed in parallel with all runs yield standard curves for AQUA normalization. (Pearson's R ranged from 0.69 to 0.82). (TIF) [file pone.0079901.s002.tif]
